# Magnetic stimulation in the treatment of female urgency urinary incontinence: a systematic review

**DOI:** 10.1007/s00192-023-05492-7

**Published:** 2023-03-06

**Authors:** Anja Antić, Maja Pavčnik, Adolf Lukanović, Miha Matjašič, David Lukanović

**Affiliations:** 1 Faculty of Medicine, University of Ljubljana, Ljubljana, Slovenia; 2 Division of Gynecology and Obstetrics, Ljubljana University Medical Center, Ljubljana, Slovenia; 3 Department of Gynecology and Obstetrics, Faculty of Medicine, University of Ljubljana, Ljubljana, Slovenia; 4 Department of Education Studies, Faculty of Education, University of Ljubljana, Ljubljana, Slovenia

**Keywords:** Magnetic stimulation, Urinary incontinence, Urgency urinary incontinence

## Abstract

**Introduction and hypothesis:**

This systematic review analyzes published studies about magnetic stimulation (MS) treatment for UUI and determines whether this treatment is effective and non-invasive.

**Methods:**

A systematic literature search was conducted using PubMed, the Cochrane Library, and Embase. The international standard for reporting results of systematic reviews and meta-analyses (Preferred Reporting Items for Systematic Reviews and Meta-Analyses) was used to guide the methodology of this systematic review. The key search terms were as follows: “magnetic stimulation” and “urinary incontinence.” We limited the time frame to articles published from 1998, when the FDA approved MS as a conservative treatment option for UI. The last search was performed on 5 August 2022.

**Results:**

Two authors independently reviewed 234 article titles and abstracts, of which only 5 fitted the inclusion criteria. All 5 studies included women with UUI, but every study had different diagnostic and entry criteria for patients. They also differed in their treatment regimens and methodological approaches to assessing the efficacy of treating UUI with MS, which made it impossible to compare the results. Nonetheless, all five studies established that MS is an effective and non-invasive way of treating UUI.

**Conclusions:**

The systematic literature review led to the conclusion that MS is an effective and conservative way of treating UUI. Despite this, literature in this area is lacking. Further randomized controlled trials are needed, with standardized entry criteria, UUI diagnostics, MS programs, and standardized protocols to measure the efficacy of MS in UUI treatment, with a longer follow-up period for post-treatment patients.

## Introduction

Urinary incontinence (UI) is a common health, hygiene, social, societal, and economic problem [1]. Since 2002, UI has been defined by the International Continence Society (ICS) as any involuntary leakage of urine [2].

Urinary incontinence is differentiated according to the underlying pathophysiological mechanisms and is divided into the most common types: stress (SUI), urgency (UUI), mixed (MUI), and “overflow” UI [2]. This paper focuses on UUI, which is defined by the ICS as the involuntary leakage of urine through the urethra that occurs with the sensation of a sudden strong urge to urinate (i.e., urgency). UUI can be part of a larger syndrome called overactive bladder syndrome (OAB), which consists of urinary urgency, increased frequency of urination, and nocturia, with or without UUI, and without urinary tract infection or other pathological conditions [3]. When OAB is associated with UUI, it is referred to as UUI. According to EUA guidelines, a thorough baseline assessment should be carried out to classify the type and severity of symptoms and elucidate any signs of UI, associated POP, concomitant UTI, current anticholinergic burden, associated neurological dysfunction, or genitourinary symptoms of menopause [4].

Conservative approaches to UUI treatment include extracorporeal magnetic stimulation (MS), which was approved by the FDA as a treatment option for UUI as early as 1998 [5]. MS is widely offered as a treatment for UI, although weak evidence of the short-term and long-term effects has been found in systematic reviews (SRs). Moreover, current EUA recommendations from 2020 advise not offering magnetic stimulation in the treatment of UI or OAB (strength of recommendation = strong) [6].

The mechanical principle of UI therapy with MS is mostly based on Faraday’s law of induction. All the nerves, especially the muscle nerves of weakened and non-active muscles, which are the cause of UI problems, can be represented as a conductor in an alternating magnetic field. This is why the electrical current is induced on all the nerves that are located in the alternating magnetic field created by the MS device. The induced current on the nerves causes the activation of weakened or damaged muscle fibers via the sodium/potassium pump (Na^+^/K^+^-ATPase) enzyme. In the case of UUI, afferent branches of the pudendal nerve are stimulated by the alternating magnetic field to inhibit the detrusor muscle through central reflexes. Simultaneously, the efferent nerve branches are also stimulated to facilitate strengthening of the pelvic floor muscles and increase the tonus of the urethral sphincters, thereby inhibiting the detrusor muscle through the guarding reflex. Because the nerves that transmit the signal from the brain to the muscle and the nerves that transmit the signal from the muscle to the brain are simultaneously affected by the alternating magnetic field (MS) produced, we achieve a better and stronger signal on a natural biofeedback (nerve) loop, directly affecting all the nerves and muscles inside the magnetic field produced, thereby having a beneficial effect on treating the UUI problems [5, 7–10].

Treatment options for UUI are limited because both UUI and OAB are chronic conditions that vary in frequency and intensity of symptoms and signs over the course of a person's life. Therefore, a multi-stage approach to treatment is needed, because a complete cure is rare, but symptom relief can be expected. This is why MS may have a place in the treatment of UUI.

To demonstrate the prevalence of the issues stated above, a systematic literature review was conducted. The review was carried out to point out the role of magnetic stimulation in the treatment of female UUI and to comprehensively evaluate the studies of the efficacy of magnetic stimulation as a treatment of UUI. We were particularly interested in the methodological approach used by the authors in designing research on the treatment of UUI with MS. Three SRs of the literature on the efficacy of MS have been published in recent years, but all of them focused on UI in general rather than on the efficacy of MS in a specific subtype of UI (SUI, UUI, or MUI), or they devoted only a small part of the review to this topic [11–13].

## Materials and methods

The aim of this SR was to analyze published studies on MS treatment for UUI. The following research questions (Q) were addressed through the systematic literature review:

- Q1. Do the authors report statistically significant results in improving UUI with MS?
- Q2. Which methods are used for monitoring the efficacy of the MS therapy?
- Q3. Did the authors follow EUA guidelines for initial diagnostics of UUI?
- Q4. What are the limitations of the studies reviewed?
- Q5. What is the length of follow-up?

All the research questions are addressed in this article.

The international standard Preferred Reporting Items for Systematic Reviews and Meta-Analyses (PRISMA) was used to guide the methodology of this SR [14]. A systematic literature search was conducted using PubMed, the Cochrane Library, and Embase. The key search terms were “magnetic stimulation” and “urinary incontinence.” We reviewed all research articles in English published since 1998 (no upper limit). We limited the time frame to articles published since 1998, when the FDA approved MS as a conservative treatment option for UI. The last search was performed on 5 August 2022. It should also be noted that this article focuses only on research articles; to our knowledge, no volume or book chapter relies on empirical work regarding the efficacy of MS in a particular UI subtype.

**Fig. 1:**
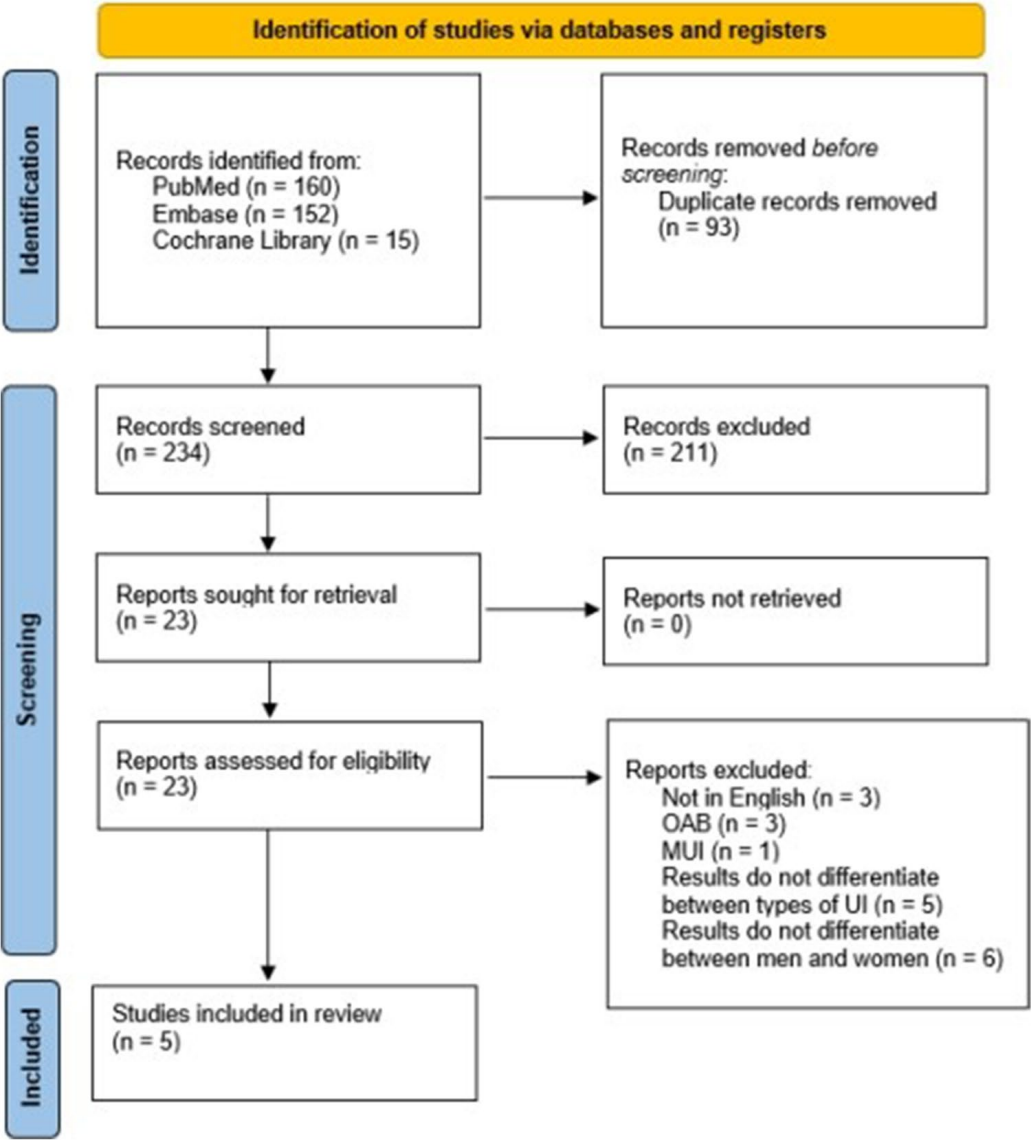
Preferred Reporting Items for Systematic Reviews and Meta-Analyses flowchart. *OAB* overactive bladder, *MUI* mixed urinary incontinence, *UI* urinary incontinence)

We identified the potentially relevant research articles by examining the abstracts or articles as a whole. Titles and/or abstracts of the studies retrieved using this search strategy and those from additional sources were screened independently by two review authors to identify studies that potentially met the inclusion criteria of this SR. We only included studies focusing on women diagnosed with UUI, in which an MS stimulator was built into a chair, and with the full text available in English. We emphasize that we wanted to include only studies with patients with UUI and not patients with full-spectrum OAB. Nonetheless, we excluded studies that did not separate the results for women and men, treated other types of UI, or used other types of MS devices (a coin, electrode, etc.), and for which the full text was not available in English. We also excluded SR, meta-analyses, clinical cases, and editors’ comments. The full texts of these potentially eligible articles were retrieved and independently assessed for eligibility by two other review team members. The PRISMA flowchart and search strategy are summarized in Fig. 1. Any disagreement between the readers regarding the eligibility of particular articles was resolved through discussion with a third (external) reviewer. Two authors independently extracted data from articles about study characteristics and outcomes. Any discrepancies were identified and resolved through discussion (with a third external reviewer where necessary). A systematic literature review was peer-reviewed by experts in the field (urogynecology consultants A.L. and M.B.) to ensure that the methods used in the review were appropriate and that the conclusions are supported by the evidence. Nevertheless, based on the literature, we followed several methods to assess the quality of our systematic literature review and the studies included; that is, we used some common methods: in addition to the PRISMA checklist, the methodological quality of the reviews included was assessed using the AMSTAR2 (A Measurement Tool to Assess Systematic Reviews) quality assessment tool, which showed moderate quality of the studies included. Moreover, we used NHLBI Study Quality Assessment Tools in order to assess the quality of the studies included [15–17]. This SR was registered in PROSPERO (no. CRD42022351055).

## Results

A total of 234 titles and abstracts were reviewed, and 211 articles did not meet inclusion criteria shown in Table 1. Furthermore, articles in which the authors included patients with UUI (different UI subtypes included, including UUI), but no separate analysis of MS success in patients with UUI was performed, were excluded. In the end, five articles that met the inclusion criteria were identified. They are presented in Table 1.

**Table 1:** Overview of studies included in this systematic review

Reference	Type of study	Equipment used	UUI patients (*n*)	Control group	Treatment regimen	UUI diagnostic methods	Methods for assessing therapy efficacy	Results (statistically significant)	Length of post-treatment follow-up	Limitations
Ünsal et al. [21]	Prospective	Neotonus Inc., (Marietta, GA, USA)	17	No	20 min MS (10 min at 5 Hz, 10 min at 10 Hz) twice a week, 8 weeks	Gynecological history and status; bladder ultrasound; urinalysis; urodynamic measurements	Three-day bladder diary; pad test; urodynamic measurements; recovery (no UI); improvement (frequency of incontinence episodes reduced by more than 50%)	Increased FDV and MCC; fewer incontinence episodes in 24 h; 3. Reduced number of daily voids	12 months	No control group; small sample size
Chandi et al. [18]	Prospective	NeoControl (Neotonus Inc., Marietta, GA, USA)	12	No	21 min MS (2 × 10 min at 10 Hz, 1-min break in between) twice a week, 8 weeks	History	Bladder diary; urodynamic measurements; pad test (at the end); VAS 5; recovery (<3 g of urine in 24-h pad test); improvement (50% or greater reduction in incontinence episodes or more than 50% fewer daily voids)	Reduced number of daily voids; less urine loss in 24-h pad test; lower VAS score	–	No control group; small sample size; no monitoring of efficacy duration after completion of therapy
Doğanay et al. [19]	Prospective	Neotonus Inc., (Marietta, GA, USA)	69	No	20 min MS (10 min at 5 Hz, 1–5 min rest, 10 min at 50 Hz) twice a week, 8 weeks	N/A	Five-day bladder diary; urodynamic measurements; I-QOL questionnaire; VAS; improvement (reduced frequency of incontinence episodes by more than 50%); recovery (no incontinence episodes)	Increased FDV and MCC; improved I-QOL results	36 months	No control group
Yamanishi et al. [20]	Multicenter, randomized, sham controlled	SMN.X (Nihon Kohden Corp., Tokyo, Japan)	151	Yes	25 min MS at 10 Hz twice a week, 6 weeks	Urinalysis; bladder diary; history	Bladder diary; OABSS questionnaire; IPSS QOL questionnaire	Reduced number of incontinence episodes per week; reduced number of voids per day; increased urine volume per void; lower IPSS QOL values	–	No urodynamic measurements; no monitoring of efficacy duration after completion of therapy
Lukanović et al. [11]	Prospective	Iskra Medical Magneto STYM (Iskra Medical d. o. o., Ljubljana, Slovenia)	23	No	20 min MS at 10 Hz every other working day for 4 weeks up to a total of 10 sessions	History and status; bladder diary; urinalysis; ICIQ-UI SF questionnaire	ICIQ-UI SF questionnaire	Lower ICIQ-UI SF values	–	No control group; no urodynamic measurements; no monitoring of efficacy duration after completion of therapy

*UUI* urgency urinary incontinence, *MS* magnetic stimulation, *UI* urinary incontinence, *FDV* first desire to void, *MCC* maximum cystometric capacity, *I-QOL* incontinence quality of life, *VAS* visual analog scale, *IPSS QOL* International Prostate Symptom Score-QoL index, *OABSS* Overactive Bladder Symptom Score, *ICIQ-UI SF* International Consultation on Incontinence Questionnaire-Urinary Incontinence Short Form

The study conducted by Yamanishi et al. was a randomized, sham-controlled trial, but the other four were prospective studies without control groups [11, 18–21]. All five studies included women with UUI, but each study used different diagnostic procedures and entry criteria for patients.

Table 1 shows that each study diagnosed UUI in a different way. Chandi et al. simply used patient histories. Yamanishi et al. added urinalysis and bladder diaries, and Lukanović et al. used all of these in addition to the ICIQ-UI SF questionnaire, which is the only one validated in Slovenian [11, 18, 20]. Ünsal et al. also used bladder ultrasound and urodynamic measurements [21]. Doğanay et al. did not especially emphasize the diagnosis of UUI [19]. The European Association of Urology (EAU) recommends that the diagnostics of treated patients should include history and status, suitable validated questionnaires, at least 3 days’ bladder diary, and urinalysis in order to rule out lower urinary tract infections. Urodynamic measurements are not indicated in uncomplicated forms of UI, because they do not affect the outcome of conservative treatment and often lead to invasive procedures [6].

Treatment regimens also differed between the clinical studies. Lukanović et al. decided on the least therapy; that is, ten sessions. Doğanay et al., Chandi et al., and Ünsal et al. selected the most sessions, that is, 16. Yamanishi et al. settled on 12 sessions [11, 18–21]. Only Lukanović et al. offered therapy every other working day; the other studies carried out therapy twice a week [11, 18–21]. The duration was relatively similar, ranging from 20 to 25 min. The magnetic field pulsation frequency also varied. Yamanishi et al., Chandi et al., and Lukanović et al. used stimulation at 10 Hz for the entire duration of therapy [11, 18, 20]. Ünsal et al. used 10 min of stimulation at 5 Hz and 10 min at 10 Hz, whereas Doğanay et al. used 10 min at 5 Hz and the other 10 min at 50 Hz [19, 21].

The studies used different criteria to determine treatment efficacy. For their main criteria, Ünsal et al. used bladder diaries, pad tests, and urodynamic measurements. All the tests showed statistically significant improvement 1 year after completing therapy. Using their definition of recovery, 6 patients (40%) were UUI symptom-free after 1 year [21]. Chandi et al. also chose to use urodynamic measurements, bladder diaries, and pad tests. They also added the visual analog scale (VAS) to assess patients’ satisfaction with therapy. The VAS values were statistically significantly higher, which indicated subjective UUI symptom improvement in participants. Significant reductions were seen in the pad tests and void frequency, whereas the urodynamic measurements did not show improvement. They argued that this could result from using pulsed magnetic fields at frequencies that are not effective for treating UUI [18]. Doğanay et al. used bladder diaries, urodynamic measurements, and the VAS for patients’ subjective assessment of therapy efficacy. They also added the Incontinence Quality of Life (I-QOL) questionnaire. Using their definition of recovery (Table 1), 40 (58%) patients recovered and 18 (26%) improved. Their urodynamic parameters increased significantly, as did their quality of life (QoL). After 6 months, symptoms recurred in 53% of patients [19]. Yamanishi et al. monitored treatment efficacy with bladder diaries, the Overactive Bladder Symptom Score questionnaire (OABSS), and the International Prostate Symptom Score QoL (IPSS QOL) index. Patients were randomized into treatment and control (sham treatment) groups by age, number of incontinence episodes at the start of the bladder diary, previous treatment attempts, and the centers where the treatments were carried out. In the bladder diaries, the number of incontinence episodes per week in the treatment group compared with the control group decreased significantly (*p*=0.038), as did the number of episodes of voiding urgency in 24 h (*p* = 0.011). A statistically significant increase in the treatment group compared with the control group was also seen in the average volume of urine excreted per void. The treatment group also had a significant drop in the average number of points on the IPSS QOL index (*p*=0.035). The average number of points on the OABSS questionnaire in the treatment group compared with the control (sham treatment) group did not significantly decrease (*p*=0.057) [22]. Lukanović et al. used the ICIQ-UI SF as their main tool for monitoring therapy efficacy. Their study included not only patients with UUI but also patients with SUI and MUI, and so separate analyses were conducted for each type. They established that the symptoms and signs of UUI improved following MS therapy. The ICIQ-UI SF results improved, regardless of patients’ UI subtype, but the best improvements in ICIQ-UI SF results were in patients with SUI. Moreover, the results show that the UI subtype had a statistically significant impact on treatment assessment in both MUI and SUI, but not UUI [11].

Table 1 presents the limitations of each study. Only Yamanishi et al. used a control group [20]. Only Doğanay et al. and Yamanishi et al. had trial samples of more than 25 patients [19, 20]. Doğanay et al. monitored therapy efficacy for 36 months, and Ünsal et al. for only 12 months [19, 21]. The others did not carry out long-term follow-up [11, 18, 20].

## Discussion

Magnetic stimulation is a method approved by the FDA as a conservative approach to treating UI, which is not believed to cause serious side effects. According to the EAU guidelines, MS is still not recommended as a treatment method owing to the lack of methodologically sound studies that scientifically evaluate findings on the efficacy and long-term effects of the treatment [4].

The systematic literature review from 1998 onward revealed only five published studies that analyzed the efficacy of MS treatment for UUI, ranging from 2003 to 2019. Only Yamanishi et al. conducted a randomized, controlled trial (RCT), but this study did show a statistically significant difference in the efficacy of MS treatment for UUI between the treatment and control (sham treatment) groups. The number of incontinence episodes per week was lower, as was the number of daily voids, and the QoL improved. The other studies had similar conclusions. Their results confirmed that MS is an effective and non-invasive way to treat UUI [11, 18–21].

Answering the research questions posed here leads to further discussion and joint conclusions. Each of the studies reviewed has its limitations, which are presented in Table 1 and are the answer to Q4. These limitations should be considered when interpreting the results published in the studies. First, and perhaps most importantly, most samples, except for the study by Yamanishi et al., were nonrandomized [20]. Although this nonprobability sampling method is the most applicable and widely used method in clinical research, the sampling method does not guarantee equal chances for each subject in the target, it is less representative of the target population, and it decreases the ability to draw completely impartial conclusions about the effectiveness of MS [22]. Second, the power of most studies in our SR was low (Table 1). An ideal study is one that has high power. This means that the study has a high chance of detecting a difference between groups if it exists, and consequently, if the study demonstrates no difference between groups, the researcher can be reasonably confident in concluding that none exists. According to the literature review, the ideal power for any study is considered 80%. For example, for the study by Lukanović et al. [11], to achieve a significance level of 95% and a power of 80%, the sample size should equal 189 [23, 24]. Only the study by Yamanishi et al. included more than 100 patients; precisely 151 [11, 20]. This means that all other studies in our SR had low power, and studies with lower power increase the likelihood that a statistically significant finding represents a false-positive result.

The studies used various means of monitoring the treatment efficacy (Q2). These results cannot be directly compared with one another even though the results, which were statistically analyzed, did indicate successful treatment of UUI with MS (Q1). As early as 1998, the International Consultation on Incontinence (ICI) and EAU recommend five domains of interest that should be reported in research studies, including patient observations, quantification of symptoms, clinician observations (anatomical, functional, compliance), QoL, and socioeconomic outcomes. Unfortunately, none of the studies reviewed reported all five domains. However, according to the last report by the EUA, questionnaires should be validated for the language in which they are being used and demonstrated to be sensitive to change [4, 25]. For example, only ICIQ-SF as a patient questionnaire for UI is available as a validated questionnaire in Slovenian, which makes it impossible for smaller countries to equally and objectively participate in measuring outcomes according to the guidelines mentioned above [26]. Moreover, there is no evidence to indicate whether the use of QoL or condition-specific questionnaires has an impact on the outcome of treatment. Therefore, it would be necessary to standardize monitoring of the efficacy of MS treatment for UUI, which would allow direct comparison between studies and define the appropriate time frame for monitoring therapy efficacy (Q5).

Single-arm clinical studies by Doğanay et al. have shown that the effects of MS continued for about a year post-treatment, but efficacy progressively diminished and came close to baseline at the 2nd and 3rd year after treatment [19]. The only RCT included in our review has no follow-up, which makes the long-term efficacy of MS for UI questionable [20].

In reviewing the studies, we found considerable variability in patient characteristics and data collected. The SR of these studies shows that it is necessary to standardize the entry criteria (Q3) and the diagnosis of UUI (Q3).

Further adding to our quandary are the poorly standardized MS protocols. To clarify the impact on the extent of amelioration after therapy with MS, the stimulation parameters should be unified with regard to time frame, impulse intensity, and follow-up tracking. A specific therapy program for different types of UI is usually suggested by producers and based on previous experience. To date, the optimal frequency and pulse duration have not yet been established, although a higher dose of 50 Hz has been reported to be the dose required to achieve good pelvic floor contraction for the treatment of SUI, and a lower dose of 10–20 Hz is required for UUI [5, 11, 13]. Moreover, the number of treatment sessions and session frequency have not been established either, which might be potential confounders contributing to the heterogeneity in studies.

In evaluating the safety of MS, most patients generally tolerated treatment well. However, this safety profile should be interpreted with caution owing to the small sample sizes of the studies included and possible under-reporting of adverse events.

This review has several strengths and weaknesses. No meta-analysis was really performed because the studies were clinically diverse, and therefore a meta-analysis may give biased results and genuine differences in effects may be obscured. A particularly important type of diversity is in the comparisons being made by the primary studies. Furthermore, the lack of a control group can limit the validity of the meta-analysis, and, as mentioned above, only one study (by Yamanishi et al.) was an RCT. For this reason, the results are presented as a narrative review with clinical outcomes. With a comprehensive search strategy, using two main repositories, we ensured that no article on our topic was neglected. We have attempted to systematically and clearly display all outcomes analyzed; however, we did not include studies that analyze the entire spectrum of OAB because we wanted to focus exclusively on UUI. Our SR was designed as a single-arm study, and so we could not compare MS therapy with other therapeutic methods.

We are aware that, considering the lack of studies of consistent RCT data for MS in UUI, further trials are warranted, and a longer follow-up period will provide more evidence to validate the effects of MS treatment. However, taking into account the limitations of our SR, the main results from the studies analyzed confirmed that MS is effective in the treatment of UUI. Another potential limitation of our SR could be that only articles published in English were included.

According to the conclusions in the studies reviewed, MS is a simple form of treatment that can help many UUI patients from the medical, social, and also financial perspective. Because it is non-invasive, it could be used as a treatment approach at the primary level of urogynecological treatment and would thus reduce the number of unnecessary invasive treatments. When adherence to “healthy habits for a healthy bladder” (behavioral therapy) proves ineffective, MS could be the next step in UUI treatment. According to the literature, the MS treatment method does not cause the patient stress because this type of treatment is comfortable, safe, and relatively painless [7, 20].

## Conclusion

This systematic literature review concludes that MS has been demonstrated to be an effective way of treating UUI that could represent part of a tiered treatment plan for UUI. Given the drawbacks of other conservative treatments, such as the side effects of pharmacotherapy and the invasiveness of electrostimulation, vaginal cones, botulinum toxin A injections, percutaneous stimulation of the posterior tibial nerve, and sacral nerve stimulation, further research on MS is warranted, considering its inherent advantages: non-invasive nature, no need to undress, patient acceptability, automatic contractions, and minimal adverse effects. In addition, the SR process offers advantages such as being comprehensive, objective, evidence-based, and transparent. These findings suggest that MS is a viable and promising treatment option for women with UUI. Taking into account all the limitations of the published studies, which were discussed in the previous section, and the answers to our research questions, it can be established that there are various methodological approaches to determining the efficacy of treating UUI with MS. Further clinical studies are needed, especially randomized control trials with comparable and relevant outcomes, as well as standardized protocols for measuring the efficacy of MS as a conservative form of UUI treatment. It must be emphasized that studies with a longer follow-up after completing MS therapy are needed. These would offer data on the long-term efficacy of MS treatment for UUI. Furthermore, patients and clinicians need more data about possible adverse events and a cost-effectiveness analysis, which will give them the opportunity to make informed choices supported by evidence.
